# Relationship between technology acceptance model, self-regulation strategies, and academic self-efficacy with academic performance and perceived learning among college students during remote education

**DOI:** 10.3389/fpsyg.2023.1227956

**Published:** 2023-08-29

**Authors:** Ricardo Navarro, Vanessa Vega, Hugo Bayona, Victor Bernal, Arlis Garcia

**Affiliations:** Department of Psychology, Pontificia Universidad Católica del Perú, Lima, Peru

**Keywords:** academic performance, perceived learning, technology acceptance, self-efficacy, engagement, remote education

## Abstract

**Introduction:**

The aim of this study was to examine the relationship between the technology acceptance model, self-regulation strategies, and academic self-efficacy with academic performance and perceived learning among college students during remote education.

**Methods:**

The participants were 301 university students from Lima. Structural equation model was used to test the proposed theoretical relationships between the variables. On the one hand, the study sought to explore the relationship between academic self-efficacy and self-regulation strategies with the technology acceptance model. On the other hand, it sought to determine whether the three dimensions of the technology acceptance model are positively related to perceived learning and academic performance.

**Results:**

The results suggest the importance of improving psychological variables such as self-efficacy and self-regulation strategies to improve the acceptance of technology, which would also improve the academic performance and perceived learning of students in a virtual environment.

**Discussion:**

The discussion highlights the significance of self-efficacy and metacognitive strategies in influencing technology perception and attitudes, ultimately impacting perceived learning and academic performance in virtual education.

## Introduction

During the years 2020–2022, the main modality of classes in Peruvian universities was virtual due to the COVID-19 pandemic. Despite the return to regular classes in mid 2022, some experts in higher education argue that remote or blended learning may continue to be offered in universities in the coming years (Navarro et al., 2021). In this sense, virtuality has led students to question the quality of their university education, describing it as “inadequate” or “insufficient.” This reality may be due to the inherent characteristics of the remote educational experience currently offered in the country. Although teachers have received training in these topics, such training has been rather superficial, and they have not necessarily had an appropriate space to develop these skills.

Remote learning, or e-learning, refers to applications and processes that use a wide range of technologies or communication devices to support teaching, learning, and assessment processes (Kumar Basak et al., 2018; Marandu et al., 2019). In theory, a distance education environment at an institution of higher education is a learning ecosystem that integrates digital technology with teaching and learning practices as part of a significant educational innovation brought on by the advancement of technology platforms (Eze et al., 2018). This environment has been growing by 15.4% on average per year, however, due to the restrictions set by the impact of COVID-19, such as the closure of schools and universities, 60% of students worldwide were forced to be a part of it (Alqahtani and Rajkhan, 2020; Radha et al., 2020). This transition can be described as abrupt and unplanned, which brought new difficulties and challenges for students (Lemay et al., 2021).

Remote education requires the use of the internet and other essential tools to generate educational materials, educate students, and manage courses in an organization (Lemay et al., 2021). Globally, universities and other higher education institutions have been investing in these essential tools, which include learning management systems (LMS), video calling systems, file hosting services, among others. Some of the most popular are Moodle, Blackboard, Teams, Zoom, Google Meet, Google Drive, etc. (Al-Kurdi et al., 2020; Al-Maroof et al., 2020; Pal and Vanijja, 2020; Alfadda and Mahdi, 2021; Alshurideh et al., 2021). However, developing countries face many challenges in the implementation of remote education, including poor internet connectivity, insufficient knowledge of the use of information and communication technologies (ICTs), and limited content development (Radha et al., 2020). Thus, in these countries adaptability to remote education has been an special challenge for university students and staff (Navarro et al., 2021).

Among Latin American countries, there is a great diversity of situations that depend on variables such as the institutional capacities for remote education and the regulatory laws. In the case of Peru and Bolivia, regulatory laws have slowed the growth to implement effective distance education, depriving universities of the immediate response capacity that was possible elsewhere (UNESCO IESALC, 2020). Similarly, Pedró (2020) suggests that, in Latin America, the application of emergency remote education would have negative results due to 3 reasons: (a) only 52% of households have broadband equipment and connectivity, (b) the supply of quality remote education seems concentrated in a few universities, and (c) teaching and student competencies are not up to the task, since in the region students have significantly lower levels of self-regulation and discipline competencies that are essential for the success of a distance education program (UNESCO IESALC, 2020).

Navarro et al. (2021) also add the low levels of digital skills of both teachers and students, which affect academic performance and raise doubts about the competencies of university students who have been studying virtually for 2 years. In addition, the pandemic has brought about a change in learning methodologies that both teachers and students had not foreseen and were not accustomed to. This includes a new virtual learning environment where the student must play a more autonomous role than in traditional face-to-face teaching (Navarro et al., 2021; Iqbal et al., 2022). For this, it is essential that the student formulates goals, self-regulates and evaluates his learning process, and enhances his skills to work in a team, being necessary that his actions, behaviors, motivations and study habits are consistent with the role he plays (Iqbal et al., 2022).

Given this difficult context, it is relevant to explore which variables are associated with educational performance and perceived learning in university students who have attended distance education. In the international context, meta-analytic research has found several variables that predict academic performance in higher education, classifying them into psychological, individual, instructional, family, economic and technological factors (Schneider and Preckel, 2017; MacCann et al., 2020; Kocak et al., 2021). Among these variables, academic self-efficacy and self-regulation strategies have been identified as relevant factors (Van Alten et al., 2019; Strelan et al., 2020; Kocak et al., 2021), in addition to the acceptance of new technologies applied in distance education.

The Technology Acceptance Model (TAM) (Davis et al., 1989) has attempted to explain the acceptance of the use of information systems based on the relationship between two main factors: (a) Perceived Usefulness (PU), which measures the degree to which a person believes that using a given technology will improve their job performance, and (b) Perceived Ease of Use (PEOU), which indicates the degree to which a person believes that using a technology will result in less effort to perform their tasks (Yong et al., 2010). According to Davis et al. (1989), the purpose of TAM is to explain the causes of technology acceptance by users. Thus, PU and PEOU are posited as critical factors in determining a user’s intention to use an information system (Davis, 1989; Davis et al., 1989; Sharp, 2007).

Also, as part of the development and extension of TAM, new factors and variables have been incorporated, such as (c) Attitudes toward use (AU) (Davis et al., 1989; Marangunić and Granić, 2015). Unlike previous models that work with behavioral intention, such as the Theory of Reasoned Action (TRA; Fishbein and Ajzen, 1975) or the Theory of Planned Behavior (TPB; Ajzen, 1991), intention of use within the TAM would be directly influenced by PU (Davis et al., 1989). In this sense, it was suggested that there are cases where, when a system is perceived as useful, the user can form a strong intention to use it without having to form an attitude toward the use of it (Marangunić and Granić, 2015). In addition, external variables that can influence a person’s beliefs about a system are also considered (Granić and Marangunić, 2019). Such variables typically include system characteristics, user training, user involvement in system design, and the nature of the system implementation process (Venkatesh and Davis, 1996).

In line with the above, it has been identified that the use of technologies in higher education can be directly influenced by PU and PEOU (Al-Kurdi et al., 2020; Al-Maroof et al., 2020; Reddy et al., 2021; Chaveesuk and Chaiyasoonthorn, 2022). In Latin America, some research reports that PU and PEOU are associated with the acceptance and use of e-learning technologies in university students (Alshare et al., 2011; Yamakawa et al., 2013; Romero-Sánchez and Barrios, 2022). Furthermore, it is proposed that, as a result of students’ quest to adapt to online learning, there is a relationship between metacognitive self-regulation strategies, PU and PEOU (Di Gesú and González, 2021; Müller and Goldenberg, 2021; Winne and Azevedo, 2022). In order to talk about these strategies, one must first talk about self-regulated learning and learning strategies.

Learning strategies are understood as procedures and thoughts that facilitate students’ learning and, in turn, impact their academic performance (Weinstein and Mayer, 1983; Pintrich et al., 1993). In this sense, the purpose of a learning strategy is to influence how the learner selects, acquires, organizes, and integrates new knowledge (Weinstein and Mayer, 1983; Pintrich et al., 1991, 1993). Similarly, self-regulated learning involves the application of general models of regulation and self-regulation to learning contexts (Pintrich, 2000). It can be defined as an active and constructive process in which students set learning goals and attempt to monitor, regulate, and control their cognition, motivation, and behavior, guided and constrained by these goals and by the contextual characteristics of the environment in which they find themselves (Pintrich, 2000).

In addition, Pintrich (2000) proposes a structure for classifying the different stages and domains of self-regulated learning. On the one hand, there are the domains of regulation, which include cognition, motivation or affect, behavior and context; and on the other hand, there are the stages, which include anticipation, planning and activation, follow-up or monitoring, control, and reaction and reflection (Pintrich, 2000). In this line, within the different stages of self-regulated learning, there are different metacognitive self-regulation strategies: (a) the activation of metacognitive knowledge, which involves the recognition and use of tasks and cognitive strategies that are known to be useful for learning and (b) cognitive monitoring, which involves a dynamic and constant process of learning evaluation, so that the student becomes aware of whether or not he understands what he is studying, and of the strategies that have a greater or lesser impact on his learning (Pintrich, 2000).

Evidence has shown that students in remote education have chosen to adopt new cognitive and metacognitive learning strategies (Obergriesser and Stoeger, 2020; Subedi et al., 2020; Pokhrel and Chhetri, 2021; Hong et al., 2022). In this sense, it is posited that, from the implementation of new technological resources, students had to monitor, plan and evaluate the strategies they used, strengthening their metacognitive strategies (Aguilar, 2020; Sánchez-Caballé et al., 2020).

Another important construct for understanding students’ learning and engagement in an online environment is self-efficacy, as it has been found to be a significant factor in determining student intention to engage in distance education (Al-Rahmi et al., 2018; Latip et al., 2020). The concept of self-efficacy comes from Bandura’s theory (Bandura, 1986) and refers to the subject’s perception of his or her ability to perform actions and achieve specific results. More specifically, Tuckman and Monetti (2011) define self-efficacy as a person’s belief that he or she will succeed in a situation that may be difficult, which is directly related to the person’s judgment of what he or she can or cannot accomplish with his or her skills. Likewise, these beliefs are usually quite fixed, to the point that people tend to dismiss the results of their actions when there are inconsistent with them (Fernandez et al., 2017). Self-efficacy is not related to how competent a person is, but rather to how confident he or she feels in doing their job with the skills they possess, no matter how great they are. Self-efficacy believes can come through four sources: experience of success, vicarious experience, verbal persuasion, and physiological states (Rahmawati, 2019).

In an educational context, academic self-efficacy is understood as the set of each individual’s judgments about his or her own abilities to organize and execute actions required as part of managing and coping with situations related to the academic environment (Domínguez Lara et al., 2014). In this line, academic self-efficacy is considered a motivational variable because it influences the use of strategic behaviors for learning and is reinforced when the use of such strategies has positive effects for the student (Robles Mori, 2020). In an online learning environment, academic self efficacy can also be related to students beliefs about their ability to use the internet, computers, web-based learning, and instructional tools (Latip et al., 2020).

The way a student approaches different activities depends on his or her self-efficacy beliefs, since they provide a basis for evaluating the likelihood of success or failure (Robles Mori, 2020). Thus, according to Prieto (2007), a high sense of self-efficacy favors the feeling of personal security when facing certain tasks, especially when they are difficult challenges. In this sense, self-efficacy influences abilities, competence motivation and, consequently, the achievement of the task goal (Alegre, 2013). In addition, self-efficacy also influences how a student plans, organizes, and develops his or her activities because the student plans and performs according to the skills and abilities he or she believes to possess (Bandura, 1997). In relation to the aim of the present study, it was found that self-efficacy has a positive effect on the actual use of a technology, and that both PU and PEOU have a significant relationship with self-efficacy and actual use mediated by self-efficacy (Rahmawati, 2019).

Student remote educational experiences can predict their academic achievement (Chou and Liu, 2005; Kiviniemi, 2014). From the academic achievement approach, achievement is understood as the level of learning obtained by a student in a teaching-learning process and is influenced by the interaction of variables associated with the student and his or her educational context, which is expressed in a quantitative grade as a result of an evaluation (Cayuela et al., 2021). Likewise, Lamas (2015) points out that the purpose of school or academic performance is to achieve an educational goal: learning.

This type of conceptualization of academic achievement is made due to the need to identify students’ progress as well as a reflection of their learning in a simple way (Navarro, 2015; Lever et al., 2016; Navarro, 2018). Thus, several studies use grades as a manifestation of academic performance and as a dependent variable on the effect of other variables related to remote education, such as motivation toward e-learning (Torun, 2020), satisfaction with e-learning (Younas et al., 2022), e-learning readiness (Yavuzalp and Bahcivan, 2021), or e-learning strategy (Jawad and Shalash, 2020).

Finally, it was also deemed appropriate to include a measure of perceived learning for the present study, which is defined as a student’s personal judgment of knowledge and understanding of a subject (Rovai, 2002). In the international context, a review of several studies (Yunusa and Umar, 2021) has found that perceived learning in virtual higher education is predicted by several variables that can be grouped into factors like communication dynamics, the e-learning environment, the organization, and the situation and individual characteristics of the learner. Precisely among the latter, self-efficacy and academic engagement are considered predictor variables. In the Peruvian context, there is a scarcity of research on this topic.

**Figure 1:**
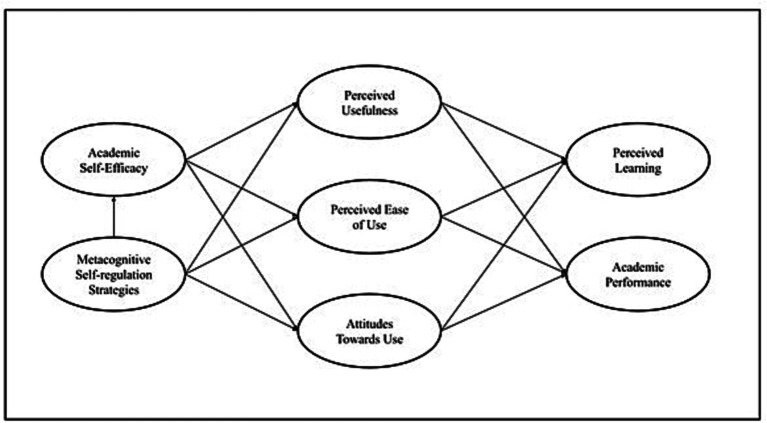
Study model.

Therefore, the present study proposes the exploration of 3 groups of variables: psychological variables (academic self-efficacy and metacognitive self-regulation strategies), technological variables (perceived usefulness of technologies, ease of use of technologies and attitudes towards the use of technologies), and educational variables (perceived learning and academic performance). In this sense, it is proposed that, from the implementation of new technological resources to carry out remote education, university students had to monitor, plan and evaluate the strategies they employed, reinforcing their metacognitive strategies (Aguilar, 2020; Sánchez-Caballé et al., 2020). Through metacognitive strategies, students select those resources and/or actions, which allow them to successfully achieve their learning goals and also influences their sense of self-efficacy (Ejubovic and Puška, 2019; Valencia-Vallejo et al., 2019; Oluwajana et al., 2021). Based on this, the present research proposes that there is a direct relationship between metacognitive self-regulation strategies and academic self-efficacy, and that these in turn directly affect technological acceptance variables (PU, PEOU, and AU), as a result of students’ quest to adapt to remote education (Di Gesú and González, 2021; Müller and Goldenberg, 2021). Finally, these variables are expected to have a direct effect on academic outcome variables (perceived learning and academic performance), being configured as mediating variables between the latter and psychological variables (see Figure 1).

## Method

### Participants

Participants were 301 college students between the ages of 18 and 32 years (M = 20.86, SD = 2.28). Regarding their gender, 173 identified themselves as female (57.5%), 124 as male (41.2%) and 4 as non-binary (1.3%). Likewise, the majority indicated belonging to the faculties of Law (19.3%), Psychology (19%), Science and Engineering (17.3%), Communications (17.2%), Arts (7.6%), Social Sciences (6.3%), Business (6%); while 7.3% indicated belonging to other faculties. Additionally, in terms of academic performance, the average grade of the participants was between 10 and 18 (M = 15.36, SD = 1.58).

The sample size was selected based on the randomness of the sample to ensure representative and valid results in order to eliminate the risk of bias. In this sense, the sample size was adequate to make representative inferences about the target population, given that it covers a range of ages (18–32 years) and a diversity in gender and faculty distribution. In addition, the variability in academic grades (M = 15.36, SD = 1.58) ensures a large and sufficient sample to obtain meaningful and generalizable results.

### Measurement

*Data sheet*. A data sheet was presented to collect the sociodemographic characteristics of the participants, such as their age, gender, university, and grade point average of the last semester, which corresponds to the study period carried out in a completely virtual manner.

*Academic self-efficacy*. The adaptation of the Perceived Self-Efficacy Scale Specific for Academic Situations (Palenzuela, 1983) was used, in its version adapted to the Peruvian university context by Dominguez et al. (2012). The scale measures the self-perceived competence in academic situation and has only one dimension, which consists of 9 items (e.g., “I consider myself qualified enough to successfully face any academic task”) and has a Likert response scale from 1 to 4, being 1 = Never and 4 = Always. Regarding its psychometric properties, Dominguez et al. (2012) report a Cronbach’s alpha of 0.89, and a KMO of 0.94, which are adequate according to Ledesma et al. (2002). Furthermore, Dominguez et al. (2013), reported that the model obtained adequate fit indices (*χ*^2^(27) = 64.687, *p* < 0.001; CFI = 0.978, TLI = 0.969, RMSEA = 0.029, SRMR = 0.056). In the present study, the scale has the following psychometric properties (*χ*^2^(27) = 51.73, *p* < 0.001; S-B*χ*^2^ = 1.463; CFI = 0.978, TLI = 0.971, RMSEA = 0.055 (CI = 0.036–0.074), SRMR = 0.034).

*Metacognitive self-regulation strategies*. The Motivated Strategies for learning Questionnaire (MSLQ; Pintrich et al., 1991) was used in its version adapted to the Peruvian university context by Matos and Lens (2006). The scale is composed of 31 items organized into 5 dimensions (rehearsal strategies, elaboration strategies, organization strategies, critical thinking and metacognitive strategies) and has a Likert response scale from 1 to 5, being 1 = Totally false and 5 = Totally true. Given that the original authors indicate that these dimensions can be used separately, only the metacognitive self-regulation strategies dimension was used for this study. This dimension assesses the student’s ability to regulate their cognitive processes during learning and includes strategies for planning, monitoring and regulating cognitive resources. The dimension is composed of 12 items (e.g., “When I study for this course I ask questions to help me focus on what I am studying”). Regarding the psychometric properties of the scale, Matos and Lens (2006) reported that the model obtained adequate fit indices (*χ*^2^(df) = 2038.20(367), *p* < 0.001; RMSEA = 0.059; SRMR =0.043). Likewise, in the present study, the scale has the following psychometric properties: *χ*^2^(df) = 49.619(45), *p* = 0.052; S-B*χ*^2^ = 1.397; CFI = 0.985; TLI = 0.980; RMSEA = 0.037 (CI = 0.013–0.056); SRMR = 0.035.

*Acceptance of technologies*. For this study, an adaptation of the version of the Technology Acceptance Model (TAM) scale developed by Teo (2011) was made. Unlike the scale developed by Teo, which has 6 dimensions, this adaptation only has three dimensions, just like the original version of the TAM developed by Davis et al. (1989). A diferencia de la escala elaborada por Teo que tiene 6 dimensiones, esta adaptación solo consta de tres dimensiones, al igual que la versión original elaborada por Davis et al. (1989). This adaptation was evaluated by a panel of judges, and then analyzed for its statistical properties. The scale evaluates the acceptance of technologies and is composed of 12 items organized in 3 dimensions: “Perceived usefulness,” which measures the usefulness that technologies have for the participant. (e.g., “The use of technology increases efficiency”); “Ease of use,” which measures how easy it is for the participant to use technologies (e.g., “It is easy for me to be trained in the use of technology”); and “Attitudes towards use,” which measures the attitudes that participants have towards technologies (e.g., “I would love to work with technologies”). The scale has a Likert response scale from 1 to 7, being 1 = Strongly disagree and 7 = Strongly agree. Likewise, it presents adequate psychometric properties in a sample of Peruvian university students (*χ*^2^(df) = 298.42(155), *p* = <0.001; S-B*χ*^2^ = 1.418; CFI = 0.954; TLI = 0.944; RMSEA = 0.056 (CI = 0.048–0.064); SRMR = 0.062).

*Perceived learning*. The Cognitive Perceived Learning in Virtuality Questionnaire (ACP-V) was used. This questionnaire was developed for this study. It was based on the research of Rovai et al. (2009) and Sher (2009) as conceptual framework. It is unidimensional and consists of 6 items (e.g., “I have understood the main concepts of the virtual courses that I took”). The scale has a Likert response scale from 1 to 5, being 1 = Strongly disagree and 7 = Strongly agree. It was evaluated by expert judges and went through a pilot prior to its application. The corresponding modifications to the items were made based on the results of these procedures. The response option of this instrument is a Likert-type scale, where 1 = “Totally disagree” and 5 = “Totally agree.” Regarding its psychometric properties, it has a Cronbach’s alpha of 0.91 and a KMO of 0.91, which, according to Ledesma et al. (2002), are adequate. Likewise, the model has good fit indices, as proposed by different authors (Hu and Bentler, 1999; West et al., 2012): *χ*^2^(df) = 16.338(9), *p* = <0.05; S-B*χ*^2^ = 1.265; CFI = 0.990; TLI = 0.984; RMSEA = 0.052 (CI = 0.009–0.088); SRMR = 0.022.

### Procedure

The instruments were designed and administered using a virtual format that was shared through the communication channels of a private university. Participants were presented with an informed consent form, which explained the purpose of the study and the characteristics of their participation. This was followed by a sociodemographic data sheet and the questionnaires.

### Data analysis

The data were coded and analyzed in RStudio software (RStudio Team, 2020). First, the existence of missing cases and extreme values was examined. Then, descriptive statistics were calculated, and the multivariate normality assumption was evaluated using Mardia’s test (1970). Subsequently, the structural equation model was performed with the robust maximum likelihood estimation method (MLM), which is recommended to correct for the possible absence of multivariate normality (Satorra and Bentler, 2001). To evaluate the model fit, the following indices were taken into consideration: Bentler-Bonett Comparative Fit Index (CFI), Tucker-Lewis Index (TLI), root mean square error of approximation (RMSEA) and standardized root mean square residual (SRMR). According to various authors, the criteria that represent a good fit are the following: for the CFI and TLI values above 0.90; for the RMSEA values below 0.06; and for the SRMR values below 0.08 (West et al., 2012; Brown, 2015).

## Results

Prior to SEM analysis, the multivariate normality assumption was evaluated. The results of Mardia’s test suggest that the data set does not comply with this assumption, since it presents multivariate skewness (ˆγ1, *p* = 3976.559, *p* < 0.001) and kurtosis (ˆγ2, *p* = 28.669, *p* < 0.001). For that reason, when performing the SEM analysis, the maximum likelihood estimation method with the correction of Satorra and Bentler (2001) was used. Thus, the results suggest that the hypothesized model presents a good fit: *χ*^2^(df) = 898.751(646), *p* = <0.001; S-B*χ*^2^ = 1.168; CFI = 0.953, TLI = 0.949, RMSEA = 0.036 (CI = 0.031–0.041), SRMR = 0.055.

As for the regressions, Metacognitive Self-Regulation Strategies were found to predict Academic Self-Efficacy (*β* = 0.376, *p* < 0.01). It was also found that Perceived Usefulness of Technologies is predicted by Metacognitive Self-Regulation Strategies (*β* = 0.324, *p* < 0.01) and Academic Self-Efficacy, (*β* = 0.402, *p* < 0.001). Likewise, Ease of Use of Technologies was found to be predicted by Metacognitive Self-Regulation Strategies, (*β* = 0.316, *p* < 0.01) and Academic Self- Efficacy (*β* = 0.252, *p* < 0.01). In addition, a relationship was found between Attitudes Towards the use of Technologies and Metacognitive Self-Regulation Strategies (*β* = 0.327, *p* < 0.01). However, no significant relationship was found between Attitudes Towards the use of Technologies and Academic Self-efficacy (*β* = 0.116, *p* = 0.168).

**Figure 2:**
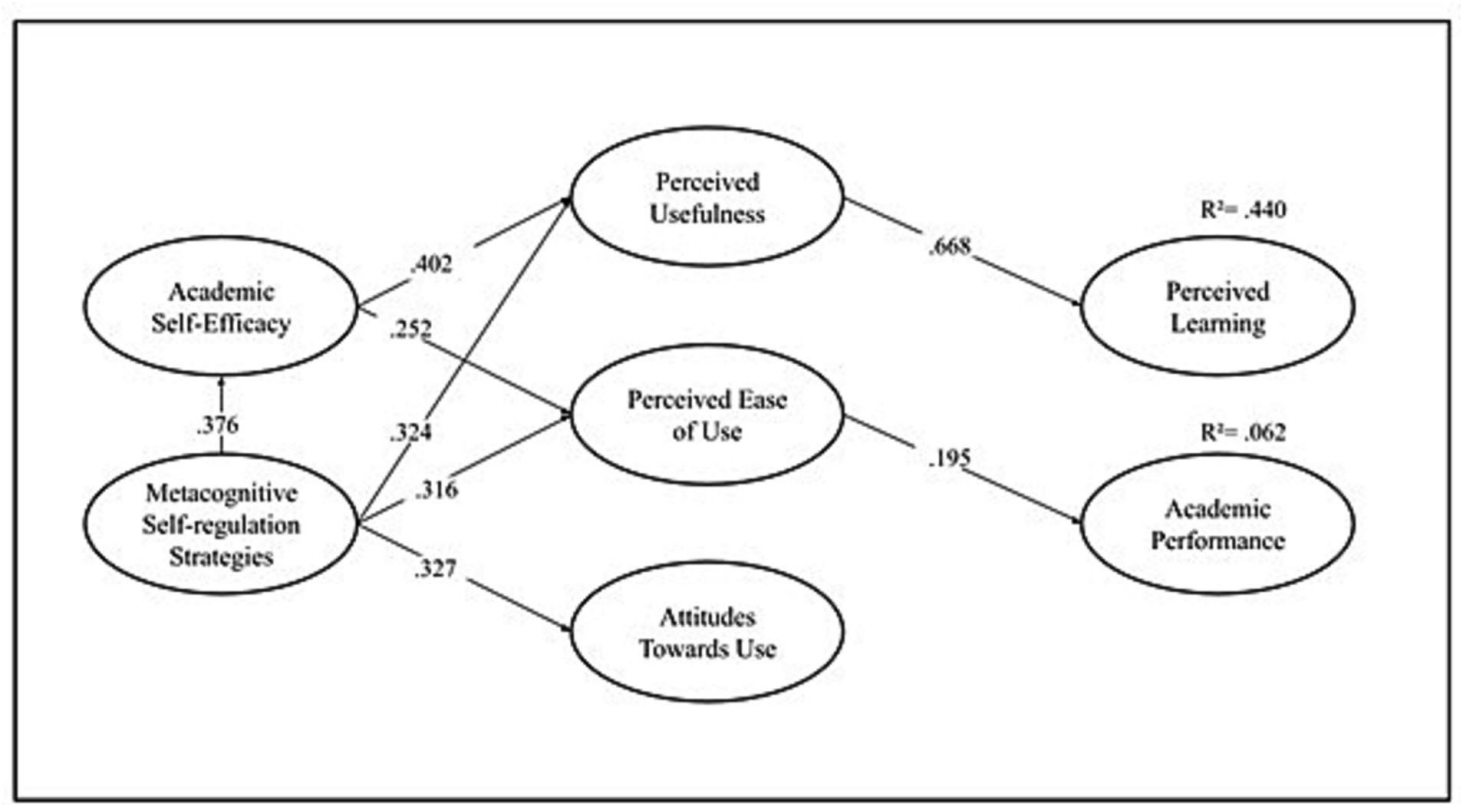
Structural equation model.

On the other hand, it was found that Perceived Learning is predicted by Perceived Usefulness of Technologies (*β* = 0.668, *p* < 0.01), but not by Ease of Use of Technologies (*β* = 0.068, *p* = 0.414), nor by Attitudes towards the use of technologies (*β* = −0.072, *p* = 0.489). Finally, Academic Performance is predicted by Ease of Use of Technologies (*β* = 0.195, *p* < 0.05), but not by Perceived Usefulness of technologies (*β* = 0.192, *p* = 0.100), nor by Attitudes towards the use of technologies (*β* = −0.169, *p* = 0.138) (see Figure 2).

## Discussion

The present study sought to explore the relationships between psychological variables (academic self-efficacy and metacognitive self-regulation strategies), technological variables (perceived usefulness of technologies, ease of use of technologies and attitudes towards the use of technologies), and educational variables (perceived learning and academic performance). All of these variables were examined in the context of the Covid-19 pandemic, which brought an abrupt and unplanned transition from face-to-face education to distance education creating new difficulties and challenges for students and teachers (Lemay et al., 2021). The findings are discussed below.

First, the relationship between metacognitive self-regulation strategies and academic self-efficacy is consistent with existing educational evidence: as students perform their academic tasks, they monitor their performance and evaluate their progress; if this is satisfactory, feelings of efficacy improve and lead students to set new challenging goals (Schunk, 1990; Blackmore et al., 2021). Thus, students who are able to identify changes in their learning over time are more likely to improve their satisfaction and self-efficacy along the way, leading to better academic performance (Zimmerman and Schunk, 2011). Similarly, when students perceive satisfactory progress toward their learning goals, they feel empowered to improve their skills and goal achievement; which also leads students to set new challenging goals (Schunk, 1990). In addition, a study conducted among users of massive open online courses (MOOCs) found a positive correlation was found between self-efficacy and the use of self-regulated learning strategies, as well as differences in the use of self-regulated learning strategies between learners who had high self-efficacy and those who had low self-efficacy (Lee et al., 2020). The same results are found in Ulfatun et al. (2021) study with virtual learners in Indonesia.

The findings regarding the direct relationship between academic self-efficacy, PU and PEOU are also in accordance with research in international contexts (Darsono, 2005; Gbongli et al., 2019; Rahmawati, 2019; Yalcin and Kutlu, 2019; Rivers, 2021). Therefore, students with a high self-efficacy tend to participate more in remote education, while those who do not believe in their ability to use technology will avoid using it to engage in class (Mallya et al., 2019). Thus, students with positive self-efficacy toward learning in remote courses tend to be more motivated and perform better in these courses (Wang et al., 2013). Therefore, higher self-efficacy can increase the acceptance of the use of a technology while lower self-efficacy can negatively affect it (Park, 2006; Alharbi and Drew, 2019). Furthermore, the findings indicate that the effect of self-efficacy is greater on PU than on PEOU, which is similar to what Teo (2009) found among students and teachers.

In the proposed model, it can be observed that PU would mediate the relationship between self-efficacy and perceived learning. There is previous evidence that self-efficacy for virtual learning is the strongest predictor of perceived learning in virtual environments (Alqurashi, 2018). This suggests that students are more likely to have a high rate of perceived learning when they attend a virtual course with high confidence in their abilities to earn a good grade, tackle difficult topics, complete online activities, and meet course expectations (Alqurashi, 2018).

Metacognitive self-regulation strategies show direct relationships with PU, PEOU, and attitudes towards the use of technologies. The latter also mediate the effect of strategies on perceived learning and academic performance. As mentioned before, self-regulation strategies involve formulating learning goals, monitoring goal achievement, and reflecting on the usefulness of strategies (Cui, 2021). With respect to remote education, prior evidence is available regarding the influence of self-regulated learning on student satisfaction and performance (Artino and McCoach, 2008; Paechter et al., 2010; Wang et al., 2013; Voils et al., 2019). There is also evidence related to the moderating effect of self-regulated learning on basic need satisfaction, positive emotions, and intrinsic motivation for learning (Cui, 2021). In addition, the mediating effect of PU and PEOU between self-regulation strategies and final intention to use has been found in previous research (Cui, 2021).

In the context of remote learning, self-regulated learning has been found to increase intention to enroll in online courses in the near future (Chen and Hwang, 2019). In addition, different studies suggested that students with low self-regulated learning skills are more likely to resist virtual courses than those with high self-regulated learning competencies (Al-Adwan et al., 2018a,b; Al-Adwan, 2020). Moreover, Al-Adwan (2020) found that the lower students’ self-regulation, the less they will recognize the value of the usefulness and functionality of virtual courses. Thus, students with a more self-regulated learning process can perform better and be more confident in dealing with the new technology involved in online courses (Chen and Hwang, 2019). Cui (2021) even mentions that the effectiveness of online learning depends more on students’ autonomous learning than on their ability to use technical equipment, so it is effective learning strategies that help adapt to learning in an emergency.

Finally, direct effects of perceived usefulness of technologies on perceived learning, and of perceived ease of use of technologies on academic performance were found. Typically, studies assessing technology acceptance consider the intention to use technology as the dependent variable, where the relationship with PU and PEOU is usually strong (Sukendro et al., 2020). In the present study, it was proposed that both variables should encourage more and better use of technology in the educational context, which should be reflected in students’ performance and perceived learning, especially in fully virtual courses. In the case of the perceived usefulness of virtual education technologies, the direct effect on perceived learning can be explained by the fact that students understand the usefulness of the technologies for their virtual classes, so they do not use them without a clear learning objective, which leads them to feel that they have a better understanding of the topics of study. On the other hand, the direct effect of ease of use on academic performance can be explained by the fact that students make good use of the technologies on which the teacher evaluates them, for example, to make a concept map or a video, which gets them a good grade, but not necessarily a learning of the course content.

## Conclusion

In conclusion, based on the analysis of the structural equation model, it was found that metacognitive self-regulation strategies predict academic self-efficacy. Also, it was found that perceived usefulness of technologies and perceived ease of use of technologies are predicted by both metacognitive self-regulation strategies and academic self-efficacy. Regarding attitudes towards the use of technologies, they are predicted only by metacognitive self-regulation strategies. Finally, it was found that perceived learning is predicted by perceived usefulness of technologies, and that academic performance is predicted by perceived ease of use of technologies.

Thus, it is recommended to foster academic self-efficacy and metacognitive self-regulation strategies in students engaged in virtual education to improve both the perceived usefulness of the technologies used and their perceived learning. Self-efficacy can be fostered through achievement, for example, through direct experiences of past success in virtual education; or through indirect experience, where students observe others successfully performing similar activities (Alqurashi, 2018). Metacognitive self-regulation strategies can be fostered through interventions in metacognition and educational resource management, in addition to informing students about effective self-regulation activities and their importance, reflecting on course material at the end of a class, and on the strategies they used to learn throughout the module (Jansen et al., 2019).

It is also observed that although significant, the effect on academic performance is low, so it is recommended to investigate what other educational variables may be involved and include them in the proposed model. Future research can be conducted considering variables such as student–content interaction, student–instructor interaction (Alqurashi, 2018), virtual classroom interaction, student motivation, course structure, instructor knowledge (Baber, 2020), or time spent on the task, cognitive activity and motivation for the task (Jansen et al., 2019). The impact of virtual instructional support, number of students enrolled in a virtual course, and teacher training should also be considered in future research (Alqurashi, 2018).

It is important to emphasize that this study used SEM for data analysis, which allows the simultaneous examination of the interaction of the variables under study, as opposed to multiple regression, which measures only the direct relationships between the independent variable and the dependent variable (Teo, 2009). In addition, the effect of technological acceptance variables on educational outcomes was found, contributing to both the technology and educational literature.

### Limitations of the study

There are also some limitations that should be considered. One limitation is that the self-report method was used for students to report the study variables, including their academic performance. Future research may seek student permission to collect this variable directly from the academic institution to reduce reporting errors. Additionally, the research focused only on the reporting of fully virtual courses, so the results cannot be generalized to hybrid or in-person courses, where the relationship between the variables may be different. In the future, a comparison between courses with different levels of virtuality can be considered to compare the results obtained (Alqurashi, 2018). Finally, demographic variables of the students, such as gender, age, career of study, previous e-learning experience, among others, can also be considered (Alqurashi, 2018), to be included within the model or to make comparisons between the study variables.

## Data availability statement

The original contributions presented in the study are included in the article/Supplementary material, further inquiries can be directed to the corresponding author.

## Author contributions

RN, VV, HB, VB, and AG contributed to conception and design of the study. RN, VB, and AG organized the database. RN, VB, and AG performed the statistical analysis. All authors contributed to writing the first draft of the manuscript, wrote sections of the manuscript, revised, read, and approved the submitted version.

## Conflict of interest

The authors declare that the research was conducted in the absence of any commercial or financial relationships that could be construed as a potential conflict of interest.

## Publisher’s note

All claims expressed in this article are solely those of the authors and do not necessarily represent those of their affiliated organizations, or those of the publisher, the editors and the reviewers. Any product that may be evaluated in this article, or claim that may be made by its manufacturer, is not guaranteed or endorsed by the publisher.
